# Supplementary material for: Mitochondrial RNA modifications shape metabolic plasticity in metastasis
Source: Nature. 2022 Jun 29;607(7919):593–603. doi: 10.1038/s41586-022-04898-5 (PMC9300468; doi:10.1038/s41586-022-04898-5)
Supplement: Supplementary file 1 — This file contains a full guide to Supplementary Tables 1–7. [file 41586_2022_4898_MOESM1_ESM.pdf]

nature portfolio

https://doi.org/10.1038/s41586-022-04898-5

Supplementary information

Mitochondrial RNA modifications shape metabolic plasticity in metastasis

In the format provided by the authors and unedited

Supplementary information guide:

Mitochondrial RNA modifications shape metabolic plasticity in metastasis

Sylvain Delaunay[1], Gloria Pascual[2], Bohai Feng[3,4], Kevin Klann[5], Mikaela Behm[1], Agnes Hotz-Wagenblatt[1], Karsten Richter[1], Karim Zaoui[3], Esther Herpel[6], Christian Münch[5], Sabine Dietmann[7], Jochen Hess[1,3], Salvador Aznar Benitah[2,8], Michaela Frye[1*]

[1] German Cancer Research Center – Deutsches Krebsforschungszentrum (DKFZ), 69120 Heidelberg, Germany; [2] Institute for Research in Biomedicine (IRB Barcelona), The Barcelona Institute of Science and Technology (BIST), 08028 Barcelona, Spain; [3] University Hospital Heidelberg, Department of Otolaryngology, Head and Neck Surgery, 69120 Heidelberg, Germany; [4] The Second Affiliated Hospital of Zhejiang University School of Medicine, Department of Otorhinolaryngology, 310014 Hangzhou, China; [5] Goethe University Frankfurt, University Hospital, Institute of Biochemistry II, 60590 Frankfurt am Main, Germany. [6] Institute of Pathology, University Hospital Heidelberg, and NCT Tissue Bank, National Center for Tumor Diseases (NCT), Heidelberg, Germany; [7] Washington University School of Medicine in St. Louis, 660 S. Euclid Ave, St. Louis, MO 63110, USA. [8] Catalan Institution for Research and Advanced Studies (ICREA), Barcelona, Spain, 08010.

* Corresponding authors: MF (M.Frye@dkfz.de)

Table of contents:

Supplementary table 1 page 3

Supplementary table 2 page 3

Supplementary table 3 page 3

Supplementary table 4 page 3

Supplementary table 5 page 4

Supplementary table 6 page 4

Supplementary table 7 page 4

Supplementary table 1: Orthotopic transplantation assays.

Overview of all orthotopic transplantation assays using human oral squamous cell carcinoma (OSCC) cells. OSCC cells were infected with control (Ctr) or two different NSUN3 (#1 and #2) shRNAs, or contained an empty vector Ctr or over-expression constructs for wild-type (WT) or mutated (MUT) enzymatic dead NSUN3. Alternatively, OSCC cells were transplanted and mice were treated with antibiotics. CTR: PBS; AMOX: Amoxicillin; DOX: Doxycycline; TIG: tigecycline. Indicated are total number of mice and mice that developed primary tumours, lymph node, and lung metastases.

Supplementary table 2: RNA-sequencing of primary tumours.

Summary table of RNA sequencing results from VDH15-derived primary tumours. VDH15 cells were infected with a control shRNA or two shRNA (#1, #2) targeting NSUN3. Shown are the $\log_2$ fold change values compared to the control shRNA (sh#2: n=4 mice/tumours; sh#1: n=3 mice/tumours). Adjusted p-Value from Deseq2 using the Wald test are shown.

Supplementary table 3: Quantitative translation measurements using mass spectrometry.

Quantitative proteomics and translation changes in CD44/CD36 high (positive) and low (negative) populations isolated from VDH01-tumouroids. Ratios between the sub-populations were calculated and significance was assessed by unpaired, two-sided Student's t test. P values were adjusted by Benjamini-Hochberg FDR correction.

Supplementary table 4: Co-translated mRNAs in CD44/CD36 subpopulations.

Genes co-translated in clusters 1-7 as shown in Figure 5j based on data shown in Supplementary Table 3. For co-expression clustering, a type II ANOVA was used on a fitted ordinary least square linear model for each protein to filter out high variance proteins. All proteins with an ANOVA P-value lower than 0.05 were used for further analysis.

Supplementary table 5: Stratification of head and neck cancer patients.

Further stratification of head and neck cancer patient cohort analysed in Figure 6. Patients are classified according to gender, smokers, alcohol consumption, HPV16-status, tumour location and size, lymph node metastases, pathological grading, and resection margin.

Supplementary table 6: Expression changes in NSUN3^{high} or NSUN3^{low} tumours.

Differential expressed genes (DEGs) between tumours of the TCGA-HNSC cohort with high or low NSUN3 expression. Shown is $\log_2$ fold change (FC), log counts per million (logCPM) and False Discovery Rate (FDR). P-value was calculated using an exact test for the negative binomial distribution.

Supplementary table 7: Molecular signature of NSUN3^{high} or NSUN3^{low} tumours.

Significant Molecular Signatures Database (MSigDB) terms for differential expressed genes (DEGs) in TCGA-HNSC cohort subgroups showing either high or low expression of NSUN3. Normalised Enrichment Score (NES) is calculated by dividing the Enrichment Score from the actual ranking by the mean of the random permutations. Random permutations (1000x) were used to calculate p-value.
