# Supplementary material for: Mitochondrial RNA modifications shape metabolic plasticity in metastasis
Source: Nature. 2022 Jun 29;607(7919):593–603. doi: 10.1038/s41586-022-04898-5 (PMC9300468; doi:10.1038/s41586-022-04898-5)
Supplement: Supplementary file 2 — Reporting Summary [file 41586_2022_4898_MOESM2_ESM.pdf]

nature research

Corresponding author(s): Michaela Frye

Last updated by author(s): May 5, 2022

Reporting Summary

Nature Research wishes to improve the reproducibility of the work that we publish. This form provides structure for consistency and transparency in reporting. For further information on Nature Research policies, see our Editorial Policies and the Editorial Policy Checklist.

Statistics

For all statistical analyses, confirm that the following items are present in the figure legend, table legend, main text, or Methods section.

n/a	Confirmed	
☐	☒	The exact sample size (*n*) for each experimental group/condition, given as a discrete number and unit of measurement
☐	☒	A statement on whether measurements were taken from distinct samples or whether the same sample was measured repeatedly
☐	☒	The statistical test(s) used AND whether they are one- or two-sided<br>*Only common tests should be described solely by name; describe more complex techniques in the Methods section.*
☒	☐	A description of all covariates tested
☐	☒	A description of any assumptions or corrections, such as tests of normality and adjustment for multiple comparisons
☐	☒	A full description of the statistical parameters including central tendency (e.g. means) or other basic estimates (e.g. regression coefficient) AND variation (e.g. standard deviation) or associated estimates of uncertainty (e.g. confidence intervals)
☐	☒	For null hypothesis testing, the test statistic (e.g. *F*, *t*, *r*) with confidence intervals, effect sizes, degrees of freedom and *P* value noted<br>*Give P values as exact values whenever suitable.*
☒	☐	For Bayesian analysis, information on the choice of priors and Markov chain Monte Carlo settings
☐	☒	For hierarchical and complex designs, identification of the appropriate level for tests and full reporting of outcomes
☐	☒	Estimates of effect sizes (e.g. Cohen's *d*, Pearson's *r*), indicating how they were calculated

Our web collection on statistics for biologists contains articles on many of the points above.

Software and code

Policy information about availability of computer code

Data collection

IVIS Spectrum bioluminescence images were acquired with Living Image v4.4 (PerkinElmer).
Confocal microscope images were acquired with LAS X v1.8.1.13759 (Leica).
Flow cytometry data were collected using BD FACSDiva 8.0.
Electron Microscopy of cell sections were observed in a Zeiss EM 910 at 120kV (Carl Zeiss, Oberkochen, Germany) and micrographs taken using a slow scan CCD camera (TRS, Moorenweis, Germany).
The collection of the oxygen consumption rate and acidification rate values were done on the software Wave Desktop 2.6.1.
For RT-qPCR, QuantStudio 5 qPCR software (Applied Biosystems) was used.
Metabolic data was acquired using Empower3 software suite (Waters).

Data analysis

Graphpad Prism 9 was used for the statistics and generation of graphs.
Flow cytometry data analysis was done with FlowJo V10 (Treestar).
Bioluminescence data were analyzed using Living Image software V4.4.
Finch TV1.5 was used to analyse bisulfite treated sequences.
Proteomics raw files were analyzed using Proteome Discoverer (PD) 2.4 software (ThermoFisher Scientific), SequestHT node and Percolator.
Gene Ontologies were determined using GSEA v4.0.3 software, ToppGene (https://toppgene.cchmc.org/), or GOrilla (http://cbl-gorilla.cs.technion.ac.il/).
Clusteranalyses was done using Clustvis 2.0 (https://biit.cs.ut.ee/clustvis/).
Quantification of signal from immunofluorescence images was done with ImageJ2 FIJI Version 2.1.0/153c.
RNAseq analysis used the following software: FastX toolkit 0.0.13; Homertools 4.7; STAR v2.3; PicardTools 1.78; featureCounts v1.4.5-p1; R v3.3.2 EdgeR v3.16; DESeq2(v1.4.1).
For in silico analyses of TCGA-HNSCC tumours, the following softwares were used: EdgeR, “GSVA” package in R, maxstat in R.
For Bisulfite sequencing analysis, the sequences were trimmed with ‘TrimGalore !’ and aligned with Bismark. The R packages RSamtools, GenomicAlignments, and VIM.

Custom perl script to compare groups in RNA-seq input table can be found here: https://zenodo.org/search?page=1&size=20&q=6518420

For manuscripts utilizing custom algorithms or software that are central to the research but not yet described in published literature, software must be made available to editors and reviewers. We strongly encourage code deposition in a community repository (e.g. GitHub). See the Nature Research guidelines for submitting code & software for further information.

Data

Policy information about availability of data

All manuscripts must include a data availability statement. This statement should provide the following information, where applicable:

- Accession codes, unique identifiers, or web links for publicly available datasets
- A list of figures that have associated raw data
- A description of any restrictions on data availability

Quantitative proteomics data are available on PRIDE (PXD021835). RNA sequencing data using VDH01 and VDH15 cells are available on EGA under the accession number EGAS00001004765 including the attached studies EGAD00001008743 and EGAD00001008742. All other sequencing data are deposited on GEO under the accession number GSE201993. Results are in part based on TCGA-HNSC (accession number phs000178) downloaded from TCGA (https://portal.gdc.cancer.gov).

Field-specific reporting

Please select the one below that is the best fit for your research. If you are not sure, read the appropriate sections before making your selection.

☒ Life sciences ☐ Behavioural & social sciences ☐ Ecological, evolutionary & environmental sciences

For a reference copy of the document with all sections, see nature.com/documents/nr-reporting-summary-flat.pdf

Life sciences study design

All studies must disclose on these points even when the disclosure is negative.

Sample size

The number of orthotopic transplantation assays per condition was chosen to be a minimum of 5 biological replicates per condition. This number is based on previous experiences in animal models and publications (e.g. Pascual et al., Nature, 2017). All experiments were generally powered to detect differences greater than 20% at a significance of $p<0.05$.
For targeted and genome-wide gene expression analyses of cultured cells, our study used a minimum of 3 replicates (one replicate = one independent infection). This sample size was estimated using previous NGS datasets (e.g. Blanco et al. 2016 Nature; Selmi et al. 2021 NAR). Flow cytometry, RT-qPCRs and seahorse assays were additionally performed in at least 3 technical replicates.

Data exclusions

No data were excluded.

Replication

All experiments were repeated at least twice and all repeats were successful.

Randomization

Experimental animals were randomly assigned to each experimental cohorts. For cell culture experiments, cells were equally distributed into multi-plate wells and the treatment condition was randomly applied. No additional controls for covariates was performed as the mice were age- and gender-matched and cells from the same passages were used for the experiments.

Blinding

Animal, cellular, flow cytometry and fluorescent quantifications were performed in a blinded manner.
Bisulfite sequenced samples were processed in a blinded manner.

Reporting for specific materials, systems and methods

We require information from authors about some types of materials, experimental systems and methods used in many studies. Here, indicate whether each material, system or method listed is relevant to your study. If you are not sure if a list item applies to your research, read the appropriate section before selecting a response.

Materials & experimental systems

n/a	Involved in the study	
☐	☒	Antibodies
☐	☒	Eukaryotic cell lines
☒	☐	Palaeontology and archaeology
☐	☒	Animals and other organisms
☐	☒	Human research participants
☒	☐	Clinical data
☒	☐	Dual use research of concern

Methods

n/a	Involved in the study	
☒	☐	ChIP-seq
☐	☒	Flow cytometry
☒	☐	MRI-based neuroimaging

Antibodies

Antibodies used

CD44 (Thermofisher, #14-0441-82, IM7, Lot#2093235 , 1:200), MTCO2 (Abcam, ab91317, Rabbit polyclonal, 1:200), MTCO1

Antibodies used	(Thermofisher, Rabbit Polyclonal, PA5-26688, Lot#SA100601BX, 1:200), cytokeratin 10 (Biolegend, PRB-159P, Poly19054, Lot#B284664, 1:200) and filaggrin (Covance, PRB-417P-100, Poly19058, Lot#B257576, 1:200), HSP90 (Santa Cruz, sc-13119, F-8, Lot#J2616, 1:1000) . NSUN3 (Genetex, GTX46175, Rabbit Polyclonal, Lot#822004446 1:100) or anti-GLUT1 (Abcam, Ab15309, Rabbit polyclonal, Lot#GR3266142-1, 1:100). The specificity of antibody staining was confirmed by IHC staining with using a rabbit IgG isotype control antibody (Cell Signaling Technology, #3900, DA1E, 1:1000). PE-Cy7-conjugated CD44 (BD Pharmingen, #560533, G44-26, Lot#0037983, 1:300), FITC- or eFluor 660- conjugated CD36 (BD Bioscience, 1:500, , #555454 and #50-0369-42, NL07, Lot#2303260, Thermofisher).
Validation	All antibodies are commercially available and have been validated in previously published studies:<br><br>Anti-CD44 (#14-0441-82, Thermofisher). This monoclonal antibody recognizes all human CD44 isoforms (clone IM7) and is reported in 213 studies. https://www.thermofisher.com/antibody/product/CD44-Antibody-clone-IM7-Monoclonal/14-0441-82<br><br>anti-MTCO2 (ab91317, Abcam). This polyclonal antibody recognizes the human MT-CO2, is validated by the manufacturer for WB application and is used in 7 different pubblished studies. https://www.abcam.com/mtco2-antibody-ab91317.html<br><br>anti-MTCO1 (PA5-26688, Thermofisher). This polyclonal antibody recognizes the human MTCO1 protein, is validated by the manufacturer for WB, IHC and immunofluorescence applications. https://www.thermofisher.com/antibody/product/MTCO1-Antibody-Polyclonal/PA5-26688<br><br>Anti-Cytokeratin 10 (PRB-159P, Bioilegend). This polyclonal antibody recognizes the human protein cytokeratin 10 and is validated by the manufacturer. https://www.biolegend.com/en-us/products/keratin-10-polyclonal-antibody-purified-10952<br><br>Anti-filaggrin (PRB-417P-100, Covance). This polyclonal antibody recognizes the human protein of filaggrin and is validated by the manufacturer. https://www.biolegend.com/en-us/products/filaggrin-polyclonal-antibody-purified-10943<br><br>anti-NSUN3 (Genetex, GTX46175). This polyclonal antibody recognizes the human protein NSUN3 and has been validated by us through NSUN3-knock-down experiments in this study and the manufacturer for WB application. https://www.genetex.com/Product/Detail/NSUN3-antibody-C-term/GTX46175<br><br>anti-GLUT1 (Abcam, Ab15309). This polyclonal antibody recognizes the human GLUT1 protein and has been used in 103 different published studies. https://www.abcam.com/glucose-transporter-glut1-antibody-ab15309.html<br><br>anti-HSP90 ((Santa Cruz, sc-13119). This monoclonal antibody recognizes the human HSP90 protein and has been used in 567 different published studies. https://www.scbt.com/fr/p/hsp-90alpha-beta-antibody-f-8<br><br>IgG isotype control antibody (DA1E, Cell Signaling Technology). This isotype control antibody are used to estimate the non specific binding of target primary antibodies due to Fc receptor binding or protein-protein interaction. It is validated by the manufacturer. https://www.cellsignal.com/products/primary-antibodies/rabbit-da1e-mab-igg-xp-isotype-control/3900?Ntk=Products&Ntt=3900<br><br>PE-Cy7-conjugated CD44 (BD Pharmingen, #560533). This conjugated antibody react with the human protein of CD44 and is validated by the manufacturer for flow cytometry. https://www.bdbiosciences.com/br/applications/research/t-cell-immunology/t-follicular-helper-tfh-cells/surface-markers/human/pe-cy7-mouse-anti-human-cd44-g44-26-also-known-as-c26/p/560533<br><br>FITC- or eFluor 660- conjugated CD36 (BD Bioscience, #555454 and 50-0369-42, Thermofisher). This conjugated antibody recognizes the human CD36 protein and is validated by the manufacturer for flow cytometry application. https://www.bdbiosciences.com/eu/applications/research/stem-cell-research/hematopoietic-stem-cell-markers/human/negative-markers/fitc-mouse-anti-human-cd36-cb38-also-known-as-nl07/p/555454. https://www.thermofisher.com/antibody/product/CD36-Antibody-clone-eBioNL07-NL07-Monoclonal/50-0369-42.

Eukaryotic cell lines

Policy information about cell lines

Cell line source(s)	SCC25, FaDu, and LentiX 293T were obtained from ATCC (https://www.lgcstandards-atcc.org). NHEK cell lines were obtained from Promocell (https://promocell.com/product/normal-human-epidermal-keratinocytes-nhek/). Biological samples to generate the patient-derived lines VDH01 and VDH15 were obtained from patients from the Hospital Vall d'Hebron (Barcelona, Spain) under informed consent and approval of the Bank of Tumour Committees of the hospital according to Spanish ethical regulations. The study followed the guidelines of the Declaration of Helsinki, and patient identity and pathological specimens remained anonymous in the context of the study.
Authentication	No other independent authentification was performed
Mycoplasma contamination	All cell lines were test for mycoplasma contamination on a regular basis. Cells used in this study were tested negative.
Commonly misidentified lines (See ICLAC register)	No commonly misidentified lines were used in this study

Animals and other organisms

Policy information about studies involving animals; ARRIVE guidelines recommended for reporting animal research

Laboratory animals	6-8 weeks old female NSG (NOD.Cg-PrkdcSCIDIl2rgtm1Wjl/SzJ) from Jackson Laboratory (Cat#0055579) were used in this study. All

Laboratory animals	animals are kept in compliance with Annex III of EU Directive 2021/63 EU from birth to death. All mice are group housed under specific pathogen-free conditions in individually ventilated cages adapted to their body weight and behavioural biology. Food, water and nesting material are provided ad libitum. Cages are placed under a 12 hours light-dark cycle and room temperature, humidity, air change/hour, and noise is maintained and monitored. None of the mice were involved in any previous procedures before the study.<br><br>Daily health checks by visual inspection were performed by the animal keepers. In case of abnormalities, individual animals are labelled as "daily observation" in the database and an automated e-mail is sent to the experimenters and the respective veterinarian/person on site to ensure that no animal is kept beyond the humane endpoints defined in the respective animal licence.
Wild animals	No wild animals were used.
Field-collected samples	No field collected samples were used.
Ethics oversight	All mice were housed in the DKFZ Central Animal Laboratory. All mouse husbandry and experiments were carried out according to the local ethics committee (DKFZ and the regional council of Baden-Württemberg state, Regierungspräsidium Karlsruhe) under the terms and condition of the animal license G-351/19.

Note that full information on the approval of the study protocol must also be provided in the manuscript.

Human research participants

Policy information about studies involving human research participants

Population characteristics	For this study tumour sections of 78 patients of the HIPO-HNC cohort were used for IHC staining. The median age at the time of diagnose was 61.1 years (range 39.7-82.5 years), most patients were males (n=62, 79.5%) and had a history of smoking (n=57, 73.1%). A history of alcohol abuse was recorded for 41 patients (52.6%), and HPV16-related tumors (n=24, 30.8%) were almost exclusively found in the subgroup of oropharyngeal squamous cell carcinoma (OPSCC). All patients were treated by surgery and most had adjuvant radiotherapy with (n=32, 41.0%) or without platinum-based chemotherapy (n=26, 33.3%) The median follow-up period was 39.93 months (range 1.73-78.27 months).
Recruitment	Patients of the Heidelberg Center for Personalized Oncology-Head and Neck Cancer (HIPO-HNC) cohort were treated between 2012 and 2016 at the University Hospital Heidelberg, Germany, and the cohort consists primarily of advanced HNSCC without evidence of distant metastasis at the time of diagnosis.
Ethics oversight	Patient samples were obtained and analyzed under protocols S-206/2011 and S-220/2016, approved by the Ethics Committee of Heidelberg University, with written informed consent from all participants. This study was conducted in accordance with the Declaration of Helsinki.

Note that full information on the approval of the study protocol must also be provided in the manuscript.

Flow Cytometry

Plots

Confirm that:

- [ ] The axis labels state the marker and fluorochrome used (e.g. CD4-FITC).
- [x] The axis scales are clearly visible. Include numbers along axes only for bottom left plot of group (a 'group' is an analysis of identical markers).
- [ ] All plots are contour plots with outliers or pseudocolor plots.
- [x] A numerical value for number of cells or percentage (with statistics) is provided.

Methodology

Sample preparation	Cells were trypsinized, washed 2 times with PBS, and fixed for 10 minutes with paraformaldehyde 1% in PBS. Following 2 additional PBS washes, cells were incubated with combinations of antibodies for 30minutes.<br>For cell sorting, cells trypsinized, washed 2 times with PBS and incubated with combinations of antibodies for 30minutes. After incubation, cells were washed twice in PBS and analyzed or flow sorted.
Instrument	BD LSRFortessaTM analyzer or a Cell sorter (BD Biosciences).
Software	FlowJo V10 software, BD FACSDiva 8.0.
Cell population abundance	The abundance of the relevant cell populations within post-sort fractions was 80-100% in experiments.
Gating strategy	Cells were first gated regarding FSC/SCC to eliminate debris, followed by FSC-H/FSC-A gating to exclude the doublet. The markers of interest were used to identify the subpopulation to analyse.<br>For FACS sorting, cells were selected on the basis of their forward and side scatter excluding cellular debris. Doublets and dead cells were eliminated by DAPI or propidium iodide.

- [x] Tick this box to confirm that a figure exemplifying the gating strategy is provided in the Supplementary Information.
